# Potassium Sulfate Spray Promotes Fruit Color Preference *via* Regulation of Pigment Profile in Litchi Pericarp

**DOI:** 10.3389/fpls.2022.925609

**Published:** 2022-06-14

**Authors:** Xuexia Su, Cuihua Bai, Xianghe Wang, Huilin Liu, Yongcong Zhu, Leping Wei, Zixiao Cui, Lixian Yao

**Affiliations:** ^1^College of Natural Resources and Environment, South China Agricultural University, Guangzhou, China; ^2^Guangdong Provincial Key Laboratory of Agricultural and Rural Pollution Abatement and Environmental Safety, Guangzhou, China; ^3^Institute of Tropical Fruit Trees, Hainan Academy of Agricultural Sciences, Haikou, China; ^4^College of Arts, South China Agricultural University, Guangzhou, China

**Keywords:** pigmentation, HSB color model, anthocyanin, pericarp acidity, foliar spray, lychee

## Abstract

Fruit color is a decisive factor in consumers’ preference. The bright red color of litchi fruit is associated with its high anthocyanin; however, poor fruit coloration is a major obstacle in litchi plantation. The role of spraying mineral nutrient salts such as KH_2_PO_4_, KCl, K_2_SO_4_, and MgSO_4_ on litchi pericarp pigmentation was examined by a field trial, and the relation between human visual color preference versus pericarp pigments and hue-saturation-brightness (HSB) color parameters was investigated. K_2_SO_4_-sprayed litchi fruit gained the maximum popularity for its attractive red color. Spray of K and Mg salts decreased the buildup of yellowish pigments, but increased the accumulation of red ones, with the exception of slightly reduced anthocyanins in KH_2_PO_4_-sprayed fruit, by regulating the activities of enzymes involved in anthocyanidin metabolism and decreasing pericarp pH, leading to varied pericarp pigment composition. K_2_SO_4_ spray generated the highest percentage of cyanidin-3-glucoside over all pigments in pericarp. Correlation analysis shows the percent of cyanidin-3-glucoside, superior to anthocyanin concentration and HSB color parameters, was a reliable indicator to fruit color preference. This work demonstrates that spray of suitable mineral salt can regulate pericarp pigment profile, and is an effective approach to improve fruit pigmentation and promote its popularity.

## Introduction

Color typically plays a vital role in the evaluation of aesthetic quality, and has been emphasized in different fields, such as psychology, physics, chemistry, optics, vision, engineering, visual arts, graphic design, urban studies, architecture, and so on (Green-Armytage, 2006). Fruit color is a pivotal commercial quality trait for fruit and a decisive factor for consumer’s preference. Fruit pigmentation can affect the taste, flavor, and smell of the fruit as well (Lewinsohn et al., 2005; Paauw et al., 2019). Pigment accumulation is responsible for fruit color (Honda and Moriya, 2018; Hu et al., 2019). Flavonoids are well recognized as the characteristic pink, red, blue, and purple anthocyanin pigments of plant tissues. For example, the major pigments present in tomatoes are the carotenoids (Chattopadhyay et al., 2021), anthocyanin accumulation is responsible for the red color of the skin and flesh of apple fruits (Honda and Moriya, 2018), carotenoids, and anthocyanin jointly contribute to the diverse colors of citrus fruits (Rodrigo et al., 2013).

Litchi (*Litchi chinensis* Sonn.), a tropical and subtropical *Sapindaceae* fruit tree with a lifespan of centuries, is widely cultivated in China and Southeast Asia. Commercial litchi plantations are developed in Africa, America, Europe, and Oceania as well. Litchi fruit is popular for its bright red color, and succulent, sweet, and unique taste and health-related nutrients (Wall, 2006; Pareek, 2016). The red color of litchi pericarp is ascribed to high concentrations of anthocyanin accumulation (Lee and Wicker, 1991; Zhang et al., 2005). However, poor and/or uneven pigmentation of fruit pericarp is a common impact in litchi production. Improvement of litchi pericarp coloration is greatly beneficial to raise the market value of litchi.

Pigmentation enhancement of fruit and vegetable before harvest is highly affected by light (Yoo et al., 2020), temperature (Balcerowicz, 2020), and their interaction (Azuma et al., 2012), as well agronomic measures such as bagging (Ma et al., 2019), fertilization (Jezek et al., 2018), and so on. These environmental factors can regulate the expression of genes in biosynthesis of pigment, leading to altered fruit pigmentation. It is widely known that litchi loses its brilliant red appearance soon at ambient condition after harvest and extensive efforts are made to solve this phenomena (Zhang et al., 2005; Fang et al., 2015), however, the role of pericarp pigmentation enhancement through foliar nutrient application preharvest is scarcely investigated in litchi. Potassium (K) is frequently supplemented to enhance the pigmentation in plants due to its multiple reactive functions in higher plants (Nguyen et al., 2010; Sulistiani et al., 2020). Thus, this study is to evaluate the role of foliar nutrient spray on pericarp pigmentation, and explore the relation between human visual color preference and pigment composition in litchi fruit pericarp, with the objective to identify an effective mineral nutrient salt to improve litchi pericarp color.

## Materials and Methods

### Nutrient Salts and Reference Materials

Four reagent grade (GR) mineral salts such as KH_2_PO_4_ (≥99.5%), KCl (≥99.5%), K_2_SO_4_ (≥99.0%), and MgSO_4_ (≥99.0%) were used as foliar nutrients in the field experiment.

All of the solvents and reagents used were GR or higher grade quality. The standard materials of gallic acid (≥99%), procyanidin B2, epicatechin (≥98%), 4-hydroxylbenzaldehyde (≥99%), rutin (≥98%), ferulic acid (≥99.5%), kaempferol-3-glucoside (97.0%), and quercetin-3-glucoside (≥98%) were purchased from Aladdin (Shanghai, China), and procyanidin A2 (≥98.0%) from Sigma–Aldrich (Saint Louis, United States). The reference materials of cyanidin-3-glucoside (≥98%) and cyanidin-3-rutinoside (≥90%) were obtained from Macklin-Lab (Shanghai, China).

### Field Experiment

A spray experiment with five treatments (four mineral salts and the control) was conducted in a commercial litchi orchard in Haikou city, Hainan province southern China. The soil in this orchard was volcanic ash soil, with a pH of 6.1. Soil alkali-hydrolyzable N, available P, and K were 325.7, 32.2, and 439.0 mg/kg, respectively. The cultivar “Ziniangxi” was used in this trial. “Ziniangxi,” a unique litchi variety called “the king of litchi” in southern China, is well accepted for its great fruit size, but simultaneously castigated by its poor pigmentation. The litchi trees were planted at the spacing of 5 m × 5 m in the spring of 2015. Twenty uniform, healthy trees were selected and divided into five groups for the spray experiment. The three K salts were sprayed at the concentration of 900 mg/L (calculated as K), and MgSO_4_ was used at the same level with K_2_SO_4_ (calculated as S). Each salt solution was sprayed to four trees, with each tree as a repeat. Spraying water was used as the control. All the solutions were evenly sprayed to the leaves and the fruits in the afternoon. There was no surfactant to be used. These trees were treated four times during fruit swelling stage from early April (35 days after fruit set) to mid-May in 2021, with intervals of 8–9 days.

### Fruit Sample Collection and Preparation

In the early morning, approximately 3 kg fruits with good quality and uniform maturity were harvested from each tree at economic ripening stage in late May. Four fruits were randomly collected from each 3 kg fruits and wrapped with paper tissues, then put into a plastic bag and sealed for color preference assessment. Then, all the fruits were immediately bagged into iced bubble chambers to keep cold, then delivered back to the laboratory on the same day by air transportation.

All the fruits for chemical analysis were rinsed with tap water and dried with a clean cotton tower in the afternoon of the same day in the laboratory. Then, the fruit epicarp (the endocarp was not included) was manually peeled and divided into four parts. The first part was frozen immediately in liquid nitrogen, and further lyophilized (Christ, Alpha 1–4 LD plus, German) and ground to a fine powder for phenolic compound detection. The epicarp powder samples were stored at −80°C till further analysis. The second part was frozen immediately in liquid nitrogen, and then stored at −80°C for enzyme activity assessment. The third part was oven-dried at 105°C for 30 min and then at 65°C to constant weight for nutrient (N, P, K, Ca, Mg, and S) analysis. The fourth part was used for epicarp pH determination immediately.

### Visual Color Preference Evaluation on Litchi Fruit

Twenty fruits from four repetitions of each treatment, were placed into a porcelain dish laid with clean white cotton cloth for color preference assessment in a well-lighted and air-conditioned room. Five dishes were prepared and aligned for the five treatments. Thirty-two participants in the age of 19–49 (students and teachers in the university, none of them was color blind) were Chinese came from all over China, half male and half female. All participants were asked to assess the fruits in five dishes and then put the rated numbers in the front of the dishes to label their preference for fruit color one by one. The five rated numbers, 1, 2, 3, 4, and 5 were used as the color preference index, referring to the first-, the second-, the third-, the fourth-, and the fifth-favorite color, respectively. The choice of each participant was recorded.

### Digital Measurement of Fruit Skin Color

After the color preference evaluation, the image of each fruit was immediately captured by a digital single-lens reflex camera (Cannon EOS-1D X, Japan). The imaging was taken under controlled and well-distributed light conditions in a mini photostudio to avoid color cast caused by environmental light. A total of 80 images were achieved. The colorimetric values of each image were extracted by Adobe Photoshop 2018 (Adobe Systems Inc., California, United States) by using hue-saturation-brightness (HSB) color model. HSB model, namely HSB space, a cylindrical coordinate system, is regarded as similar to human visual system (Jih-Gau and Yang, 2016; Agahchen and Albu, 2017), and more concordant with human perception, especially for surface with granular protuberance due to its ignorance of shading effect (Dal Grande et al., 2008). Litchi epicarp consists of tubercles, which make it more suitable for the HSB system. In HSB model, hue (H) values are defined as an angle from 0 to 360°, representing various colors. 0° stands for red, 45° refers to yellow, and 90 and 135° points to yellow-green and green, respectively. Saturation (S) is measured as a percent from 0 (white) to 100% (fully saturated color), and brightness (B) as percent from 0 (black) to 100% (fully bright color) (Cubukcu and Kahraman, 2008).

### Extraction and Detection of Phenolic Compounds in Epicarp

Approximately 0.35 g of the lyophilized epicarp powder was mixed with 7 mL of pre-frozen methyl/water (70:30, v/v, pH 0.5) solution and sonicated at 5° for 30 min, then centrifugated with 4,000 rpm at 5° for 10 min, and the supernatants were collected. The residuals were re-extracted twice with 4 mL of methyl/water solution, respectively. All the three supernatants were combined and subjected to rotary evaporation to remove the methyl, then transferred to a 25 mL volumetric flask and diluted to the constant volume with ultrapure water. The extract solution was stored at 4° and filtered with a 0.22 μm film prior to phenol detection by HPLC.

The HPLC (Agilent 1260 Infinity II Quaternary Pump) was equipped with a Caprisil C18-X column and a DAD detector (G7115A). The 2% phosphoric acid (A) and acetonitrile (B) were used as the mobile phases at the speed of 0.8 mL/min. The gradient procedure was scheduled as: 0–5 min with 10% B, 5–10 min with 10–13% B, 10–20 min with 13–18% B, 20–25 min with 18% B, 25–30 min with 18–30% B, 30–31 min with 30–50% B, and 31–33 min with 50% B.

### Enzyme Activity Essay

The activities of phenylalanine ammonialyase (PAL) and chalcone isomerase (CHI) were determined by the biochemical methods, and the kits were purchased from the Suzhou Keming Biological Technology Co., Ltd (China). PAL activity is defined as the absorbance variation of 0.1 units per mg tissue per minute in 1 mL reaction solution under 290 nm. CHI activity is measured as the absorbance variation of 0.1 units per mg tissue per hour in 1 mL reaction solution under 381 nm. The activities of both enzymes were expressed as U/g.

### Epicarp pH and Nutrients

The epicarp was manually torn apart into pieces and placed into a beaker, and ultrapure water was added (epicarp:water = 1:5, w:w). The solution was stirred for 10 min using a magnetic stirrer, and then the supernatant pH was measured by a pH meter.

The epicarp sample was digested with concentrated H_2_SO_4_ + H_2_O_2_, then N content in the digested solution was detected by Kjeldahl determination. The epicarp sample was digested with concentrated HNO_3_ + HClO_4_, and P concentration in the digested solution was determined by Mo – Sb colorimetric method, and K concentration by flame photometer, and Ca and Mg concentrations by atomic absorption spectrophotometer and S concentration by ICP-OES (Varian 710-ES, United States) (Lu, 1999). Standard materials of GBW07603 were used to assure the analysis quality.

### Data Analysis and Statistics

The mean of color preference rating score was calculated by the formula as follow.


$$\begin{array}{c} \mathrm{The}\mathrm{mean}\mathrm{of}\mathrm{color}\mathrm{preference}\mathrm{rating}\mathrm{score}=\\ \left(\mathrm{P1}*1+\mathrm{P2}*2+\mathrm{P3}*3+\mathrm{P4}*4+\mathrm{P5}*5\right)/\\ \left(\mathrm{P1}+\mathrm{P2}+\mathrm{P3}+\mathrm{P4}+\mathrm{P5}\right) \end{array}$$

Where P1, P2, P3, P4, and P5 refer to the numbers of person who give preference index 1, 2, 3, 4, and 5, respectively.

All the data are expressed as mean ± standard deviation. The color preference rating scores were compared by non-parametric analysis with Kruskal–Wallis test, and all the other data were subjected to analysis of variation, followed by Duncan’s multiple comparisons (*P* < 0.05) by SAS 9.2. The Pearson correlation analysis was conducted by SPSS 22.0.

## Results

### Fruit Peel Color Preference Rating

Among all the participants, 50% (16/32) of them chose the K_2_SO_4_-sprayed fruit as their best preferred for their more attractive and even red color, and 37.5% (12/32) and 12.5% (4/32) of the participants preferred the fruit applied with KCl and KH_2_PO_4_ over all others, respectively (Table 1). None selected the control or MgSO_4_-sprayed fruit as their favorite.

**TABLE 1:** The numbers of participants who gave their preference rating for the pericarp color of litchi fruits sprayed with K and Mg salts and the means of color preference rating score (preference index 1–5 means the popularity from the best to the least).

Preference index	Control	KH_2_PO_4_	KCl	K_2_SO_4_	MgSO_4_	Total number of person
1	0	4	12	16	0	32
2	2	5	13	9	3	32
3	3	14	7	3	5	32
4	10	6	0	4	12	32
5	17	3	0	0	12	32
Mean of color preference rating score	4.3	3.0	1.8	1.8	4.0	32

For preference rating scores, a significant discrepancy was observed among treatments (*p* < 0.0001). KCl- and K_2_SO_4_-sprayed fruits were calculated with similar preference scores (1.8 and 1.8, *p* = 0.9541), which were significantly higher than that of KH_2_PO_4_-treated fruit (*p* < 0.0001). The control- and MgSO_4_-sprayed fruits scored similarly (4.3 and 4.0, *p* < 0.1961) as well, and were significantly lower than those applied with KH_2_PO_4_ (3.0, *p* = 0.005). Totally, the control fruit was the least preferred by all the participants. The above indicates that spraying K salts, in particular K_2_SO_4_ was an effective approach to improve red color development in litchi fruit pericarp.

### Color Parameters of Fruit Epicarp

Although it is well recognized that hue, saturation, and brightness positively affect human preference for color (Camgoz et al., 2002; Wilms and Oberfeld, 2018), frequently, hue is still the dominant for color choice, regardless of saturation and brightness (Camgoz et al., 2002; Cubukcu and Kahraman, 2008; Fortmann-Roe, 2013). Traditionally, commercially ripe litchi is highly recognized for its red fruit pericarp in China and Southeast Asia. No significant difference was observed for H and S values, and significant variation was found only for B values of litchi fruit pericarp among treatments (*p* < 0.05) (Table 2). However, K_2_SO_4_-sprayed fruit was characterized by the lowest H, S, and B values, indicating that hue might be the predominant factor for color preference in litchi fruit because the lower H value represents a redder color.

**TABLE 2:** Pericarp hue (H), saturation (S), and brightness (B) values of litchi fruits sprayed with K and Mg salts and the visible color simulated by hue-saturation-brightness (HSB) values.

Treatment	*H*	*S*	*B*	Simulative visible color
Control	13.2 ± 4.3a	85.0 ± 2.1a	68.5 ± 3.4a	
KH_2_PO_4_	13.7 ± 2.9a	83.8 ± 2.6a	64.3 ± 4.4*ab*	
KCl	11.5 ± 4.0a	84.6 ± 2.0a	61.9 ± 7.2*ab*	
K_2_SO_4_	9.4 ± 5.3a	83.5 ± 4.9a	56.8 ± 6.8b	
MgSO_4_	10.4 ± 3.1a	84.0 ± 2.2a	60.4 ± 4.6b	

*Data in the row followed by different lowercase letters are significantly different at 0.05 level.*

### Phenolic Compounds in Litchi Epicarp

Five classes of phenolic compounds were identified in litchi epicarp (Table 3). Colorless flavanols such as epicatechin, procyanidin A2, and procyanidin B2 were the main phenolic compounds, with the concentrations ranging from 9241.7 ± 1097.5 to 11100.4 ± 700.7, 5959.9 ± 1106.2 to 8224.2 ± 369.3, and 2709.0 ± 294.9 to 3421.7 ± 49.3 mg/kg, respectively. Yellowish flavonols like rutin (613.5 ± 106.9–768.9 ± 50.1 mg/kg), quercetin-3-glucoside (103.2 ± 11.8–142.4 ± 5.8 mg/kg) and kaempferol-3-glucoside (43.8 ± 9.4–69.3 ± 1.3 mg/kg), and red pigments including cyanidin-3-glucoside (593.6 ± 140.6–1,194.1 ± 352.3 mg/kg) and cyanidin-3-O-rutinoside (1856.8 ± 221.2–4007.0 ± 1261.3 mg/kg) were determined. Cyanidin-3-galactoside appeared as a weak peak but could not be quantitatively determined in this study. Low levels of phenolic acids such as ferulic acid (98.4 ± 18.8–222.9 ± 61.6 mg/kg) and gallic acid and other phenol like 4-hydroxybenzaldehyde were observed as well. The primary phenol compounds were epicatechin and procyanidin A2 and the largest anthocyanin component was cyanidin-3-rutinoside in “Ziniangxi” fruit epicarp, which is in line with the observations in “Hong Huey” and “Chacapat” litchi (Reichel et al., 2011).

**TABLE 3:** Profile of phenolic compounds in epicarp of litchi fruits sprayed with K and Mg salts (Unit: mg/kg).

Phenolic compound		Control	KH_2_PO_4_	KCl	K_2_SO_4_	MgSO_4_
Flavanols	Epicatechin	11100.4 ± 700.7a	10151.2 ± 1502.8*ab*	9555.1 ± 279.1*ab*	9241.7 ± 1097.5b	9264.2 ± 742.7b
	Procyanidin A2	8224.2 ± 369.3a	7309.0 ± 915.0*ab*	6527.3 ± 497.8b	5959.9 ± 1106.2b	6179.9 ± 1151.8b
	Procyanidin B2	3421.7 ± 49.3a	3163.7 ± 664.2*ab*	2859.5 ± 217.0*ab*	2859.1 ± 422.1*ab*	2709.0 ± 294.9b
Flavonols	Rutin	768.9 ± 50.1a	613.5 ± 106.9b	710.0 ± 91.2*ab*	718.9 ± 96.5*ab*	668.7 ± 76.7*ab*
	Quercetin-3-glucoside	142.4 ± 5.8a	103.2 ± 11.8b	110.5 ± 13.1b	108.9 ± 7.6b	107.1 ± 14.3b
	Kaempferol-3-glucoside	69.3 ± 1.3a	43.8 ± 9.4c	65.0 ± 10.0*ab*	65.1 ± 17.0*ab*	47.4 ± 6.5*bc*
Anthocyanins	Cyanidin-3-glucoside	655.2 ± 165.4b	593.6 ± 140.6b	819.0 ± 284.1*ab*	1194.1 ± 352.3a	674.1 ± 162.3b
	Cyanidin-3-O-rutinoside	2060.3 ± 276.9b	1856.8 ± 221.2b	2523.9 ± 666.2b	4007.0 ± 1261.3a	2426.0 ± 462.7b
Phenolic acids	Ferulic acid	175.2 ± 9.2a	98.4 ± 18.8b	201.2 ± 40.0a	222.9 ± 61.6a	118.4 ± 16.2b
	Gallic acid	24.7 ± 3.4a	31.0 ± 9.6a	28.6 ± 3.6a	26.2 ± 5.4a	31.3 ± 5.2a
Other	4-Hydroxybenzaldehyde	99.0 ± 2.8a	96.2 ± 5.1*ab*	96.1 ± 1.8*ab*	93.3 ± 2.5b	94.4 ± 2.9*ab*
Total		26741.4 ± 1051.42a	24060.5 ± 3175.3*ab*	23496.3 ± 1609.2*ab*	24497.2 ± 2953.8*ab*	22320.4 ± 1826.6*s*b

*Data in the row followed by different lowercase letters are significantly different at 0.05 level.*

A significant difference for the concentrations of all the detectable phenols, with the exception of gallic acid, was observed among treatments (*p* < 0.05). Spraying K and Mg salts, K_2_SO_4_ and MgSO_4_ in particular, significantly decreased epicarp flavanols compared to the control. Totally, the values of flavonols were reduced by the spray of the four salts as well. Spraying KH_2_PO_4_ significantly decreased rutin content in epicarp, and supplement of all the four salts significantly reduced epicarp quercetin-3-glucoside (*p* < 0.05). KH_2_PO_4_, superior to MgSO_4_, significantly reduced kaempferol-3-glucoside in epicarp (*p* < 0.05), while KCl and K_2_SO_4_ decreased it insignificantly. In contrast to the control, spraying K_2_SO_4_ significantly increased the values of both cyanidin-3-glucoside and cyanidin-3-O-rutinoside by approximately 2-folds (*p* < 0.05), while KCl spray insignificantly raised them. Spraying MgSO_4_ and KH_2_PO_4_ slightly elevated or reduced both derivates of cyanidin, respectively. The application of K_2_SO_4_ and KCl insignificantly increased ferulic acid in epicarp, whereas both KH_2_PO_4_ and MgSO_4_ supplement, significantly decreased it (*p* < 0.05). Epicarp 4-hydroxybenzaldehyde was reduced by spray of the four salts. Generally, the total phenolic compounds were decreased by foliar spray of the four salts, MgSO_4_ in particular.

**FIGURE 1:**
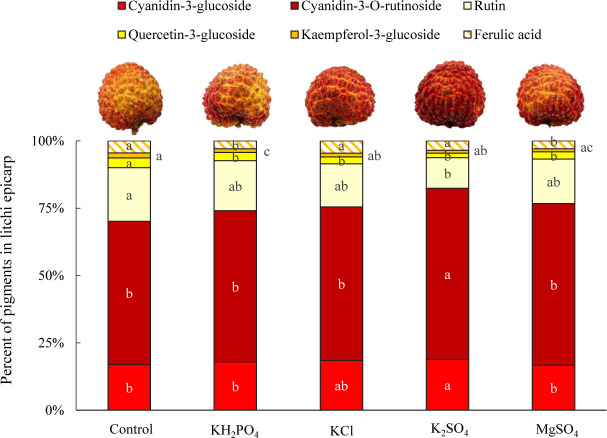
Distribution of pigments in epicarp of litchi fruits sprayed with K and Mg salts. The different lowercase letters in the bars refer to significant at 0.05 level.

Flavonols contribute to pale yellow to dark brown color, and anthocyanins endow colors from pink through red and to purple in a range of plants. The amalgamation of pigment endues versatile colors for plants. The pigment profile shows that although all the treated litchi fruits were characterized by a red tone in the epicarp; spray of K and Mg salts did alter the distribution of visible pigments in the epicarp (Figure 1). The allotment of cyanidin-3-glucoside with bright red color among treatments decreased in the order: K_2_SO_4_ (18.9%) > KCl (18.5%) > KH_2_PO_4_ (17.9%) > control (16.9%) > MgSO_4_ (16.7%), and that of cyanidin-3-O-rutinoside with dark red color was K_2_SO_4_ (63.4%) > MgSO_4_ (60.0%) > KCl (57.0%) > KH_2_PO_4_ (56.1%) > control (53.2%). Similarly, the allocation of yellowish pigments in the epicarp was decreased by the spray of the four salts. Rutin, an extremely pale yellow pigment, was the largest ingredient of the yellow-hue pigments in litchi fruit epicarp. The K_2_SO_4_ spray generated the lowest percentage of rutin (11.4%), followed by KCl (16.0%) and MgSO_4_ (16.5%). Further, K_2_SO_4_ spray reduced the allotment of quercetin-3-glucoside and kaempferol-3-glucoside as well.

### Activities of Enzymes Involving in Anthocyanin Synthesis

**FIGURE 2:**
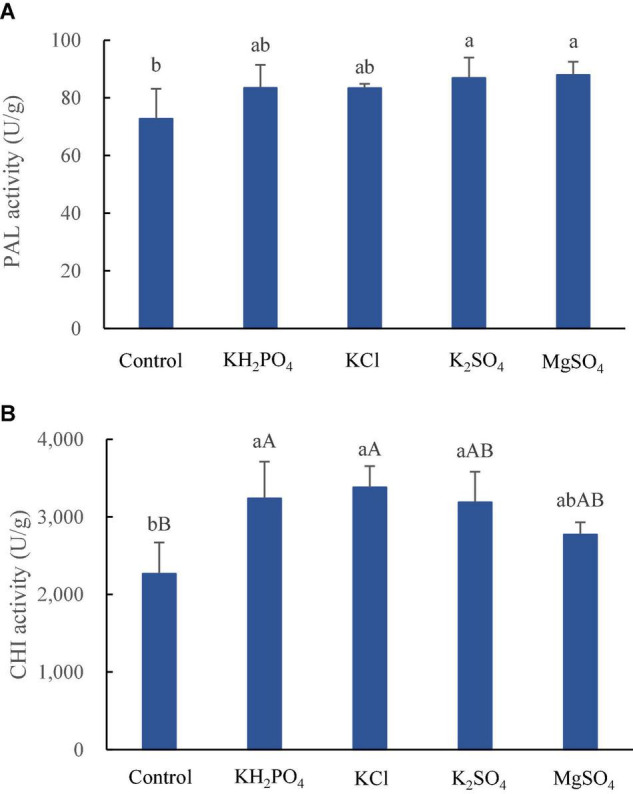
Activities of PAL **(A)** and CHI **(B)** in epicarp of litchi fruits sprayed with K and Mg salts. The different lowercase and uppercase letters attached to the bars are significantly different at 0.05 and 0.01 levels, respectively.

Phenylalanine ammonialyase is the first key enzyme in the phenylpropanoid pathway and plays a vital role in the synthesis of anthocyanins (Boudet, 2007). The expression of PAL increases from green to yellow and to red stages in litchi (Zhao et al., 2012), and epicatechin content, regulated by PAL activity, decreases during litchi fruit development (Sun et al., 2009) due to its polymerization to procyanidins (Liu et al., 2007). CHI is the key enzyme involved in anthocyanin biosynthesis in litchi pericarp as well (Qu et al., 2021). The spray of K and Mg salts, K_2_SO_4_ and MgSO_4_ in particular, significantly increased PAL activity (*p* < 0.05) (Figure 2A), which might lead to enhanced biosynthesis of epicatechin. Meanwhile, the application of the three K salts, superior to MgSO_4_ spray, significantly raised CHI activity (Figure 2B), which might promote the synthesis of cyanidins. However, epicatechin and both procyanidin derivates were decreased by spraying all the K and Mg salts (Table 3), irrespective of increased PAL activity. The discrepancy between increased PAL activity and decreased epicatechin and procyanidins might be explained that although more precursors of procyanidins were synthesized by increased PAL activity, and more of them were transformed to anthocyanidins owing to increased CHI activity, leading to lower accumulation of flavanols and higher buildup of cyanidins in litchi pericarp in the present study. The metabolism of colorants in litchi pericarp is not completely illustrated yet (Sun et al., 2009; Qu et al., 2021); however, it is well recognized that the color of litchi pericarp is determined by both synthesis and degradation or conversion of pigments; therefore, the phenolic profile in epicarp of litchi fruit sprayed with K and Mg salts is the joint effect of K and Mg salts on phenolic metabolism.

### Epicarp pH and Nutrients

**FIGURE 3:**
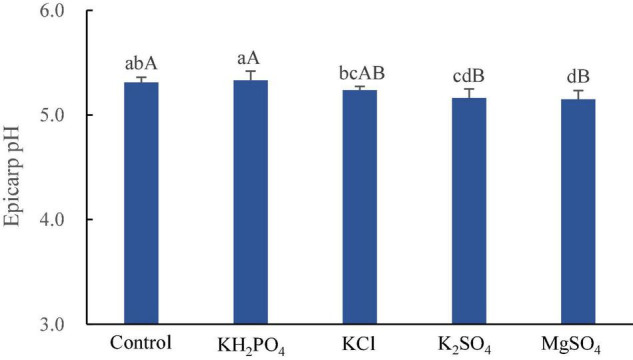
Epicarp pH of litchi fruits sprayed with K and Mg salts. The different lowercase and uppercase letters attached to the bars are significantly different at 0.05 and 0.01 levels, respectively.

The spray of KH_2_PO_4_ increased litchi epicarp pH, whereas both MgSO_4_ and K_2_SO_4_ supplement significantly decreased it (*p* < 0.01), and KCl spray reduced it insignificantly (Figure 3).

The response of epicarp nutrient to spray of K and Mg salts differed greatly (Table 4), which was probably associated with varied mobility of mineral nutrients from pericarp to pulp and then to seed in litchi (Su et al., 2022). Epicarp N, K, and S in litchi fruits were slightly raised by spray of K and Mg salts, in contrast to the control. Spray of KH_2_PO_4_ and KCl significantly enhanced epicarp P (*p* < 0.05), and K_2_SO_4_ spray increased it insignificantly, while MgSO_4_ spray did not affect it. Spray of all K and Mg salts, with the exception of K_2_SO_4_, significantly increased epicarp Ca (*p* < 0.01). Spray of the three K salts had no effect on epicarp Mg and S, whereas MgSO_4_ spray slightly increased them.

**TABLE 4:** Mineral nutrient concentrations in epicarp of litchi fruits sprayed with K and Mg salts.

Treatment	N (g/kg)	P (g/kg)	K (g/kg)	Ca (g/kg)	Mg (g/kg)	S (mg/kg)
Control	8.6 ± 0.4a	0.74 ± 0.12cB	9.0 ± 0.5a	3.68 ± 0.88bB	2.68 ± 0.19a	903 ± 41a
KH_2_PO_4_	9.6 ± 0.8a	0.83 ± 0.14aa	9.5 ± 0.3a	4.93 ± 0.37aa	2.74 ± 0.19a	924 ± 55a
KCl	10.0 ± 0.7a	0.81 ± 0.04abab	9.3 ± 0.6a	5.49 ± 0.27aa	2.68 ± 0.18a	944 ± 82a
K_2_SO_4_	9.3 ± 0.7a	0.77 ± 0.01 bcab	9.5 ± 0.7a	3.50 ± 0.65bB	2.66 ± 0.05a	937 ± 39a
MgSO_4_	9.1 ± 0.7a	0.73 ± 0.10 cB	9.5 ± 0.6a	5.16 ± 0.72aa	2.81 ± 0.09a	985 ± 45a

*Data in the row followed by different lowercase and uppercase letters are significantly different at 0.05 and 0.01 levels, respectively.*

## Discussion

### Relation Between Visual Color Preference and Pigment Palette and pH in Litchi Fruit Skin

The attractive red color of litchi fruit is believed to be ascribed to high contents of anthocyanins (Lee and Wicker, 1991; Zhang et al., 2004). Amazingly, the color preference rating score was solely correlated to the percentage of cyanidin-3-glucoside over total visible pigments in the epicarp (*r* = −0.973^**^, *p* = 0.005) in the present study (Table 5). K_2_SO_4_ spray raised not only the concentration of cyanidin-3-glucoside, but also its allotment over total visible pigments, leading to a more attractive red color.

**TABLE 5:** Pearson correlation coefficients between visual pericarp color preference rating score versus epicarp phenol level, percent of a pigment over total visible pigment and HSB color parameter in litchi fruits sprayed with K and Mg salts (*n* = 5).

Item	Correlation efficient	*P* value
Color preference rating score	1	
Epicatechin	0.587	0.298
Procyanidin A2	0.575	0.311
Procyanidin B2	0.440	0.459
Rutin	0.084	0.893
Quercetin-3-glucoside	0.539	0.311
Kaempferol-3-glucoside	–0.234	0.705
Cyanidin-3-glucoside	–0.710	0.179
Cyanidin-3-O-rutinoside	–0.582	0.303
Ferulic acid	–0.518	0.372
Gallic acid	0.035	0.955
4-Hydroxybenzaldehyde	0.529	0.359
Red pigment *^a^*	–0.642	0.242
Yellow pigment *^b^*	–0.161	0.796
Percent of cyanidin-3-glucoside	−0.973**	0.005
Percent of cyanidin-3-O-rutinoside	–0.518	0.372
Percent of rutin	0.730	0.162
Percent of ferulic acid	–0.131	0.834
Percent of quercetin-3-glucoside	0.785	0.115
Percent of kaempferol-3-glucoside	0.470	0.425
H	0.424	0.477
S	0.416	0.486
B	0.642	0.243

** Indicates significant at 0.01 level by two-tail test.

*^a^Red pigment = Cyanidin-3-glucoside + Cyanidin-3-O-rutinoside. ^b^Yellow pigment = Rutin + Ferulic acid + quercetin-3-glucoside + kaempferol-3-glucoside.*

Intriguingly, MgSO_4_-treated fruit has a higher skin H value than K_2_SO_4_-treated one but lower than KCl-treated one, but none of the participants make it as their best choice and only a fewer of them take it the second-like, leading to a similar preference rating score as the control fruit. The lower preference rating score of MgSO_4_-sprayed fruit with lower fruit pericarp H value, is probably associated with the lowest percentage of cyanidin-3-glucoside in the visible pigments (Figure 1). And the low epicarp H value in MgSO_4_-sprayed fruit might be ascribed to the high percentage of cyanidin-3-O-rutinoside with dark red color.

In addition, despite the advantage of the HSB color model, the human eye can sense the trace difference of pigment palette in litchi pericarp more subtly than a digital camera in the current investigation, as demonstrated by the discrepancies between the simulative pericarp color (Table 2) and the actual fruit color (Figure 1). It implies that the allotment of cyanidin-3-glucoside, rather than anthocyanin concentration or the HSB color parameter, is a more reliable indicator for visual color preference.

Correlation analysis shows that both derivatives of cyanidin were negatively correlated with epicarp pH (*p* < 0.05) (Table 6). The protection of red pigment in litchi pericarp conferred by low acidity (Zhang et al., 2005; Fang et al., 2013) and the color enhancement effect on cyanidin-3-glucoside by low acidity of plant cell sap (Mizuno et al., 2019) have been documented, therefore, the decreased epicarp pH in K_2_SO_4_-sprayed fruit promotes the display of red color of litchi fruit. Thus, the high percent of cyanidin-3-glucoside in epicarp and pigmentation enhancement by low epicarp pH, are jointly responsible for the popularity of K_2_SO_4_-sprayed fruit in the color preference evaluation.

**TABLE 6:** Pearson correlation coefficients between epicarp phenol concentration versus pH and mineral nutrient level in litchi fruits sprayed with K and Mg salts (*n* = 20).

Phenol	pH	N	P	K	Ca	Mg	S
Epicatechin	0.081	−0.500*	–0.326	–0.167	–0.187	–0.035	−0.243
Procyanidin A2	0.310	–0.367	–0.060	–0.225	–0.282	–0.070	−0.229
Procyanidin B2	0.036	−0.475*	–0.166	–0.153	–0.268	–0.259	−0.340
Rutin	–0.169	–0.146	0.166	–0.156	−0.479*	−0.585**	−0.239
Quercetin-3-glucoside	0.123	–0.048	0.159	0.000	–0.441	−0.502*	−0.356
Kaempferol-3-glucoside	0.050	–0.106	0.203	–0.128	−0.460*	−0.568**	−0.383
Cyanidin-3-glucoside	−0.473*	–0.075	0.055	–0.268	–0.356	–0.199	−0.180
Cyanidin-3-O-rutinoside	−0.536*	0.018	0.136	–0.300	–0.413	–0.272	−0.165
Ferulic acid	–0.225	–0.058	0.203	–0.256	−0.480*	−0.583**	−0.372
Gallic acid	0.099	0.275	0.137	0.133	0.102	0.206	−0.097
4-Hydroxybenzaldehyde	0.145	–0.117	0.226	0.062	0.072	–0.227	−0.212

* and ** indicate significance at 0.05 and 0.01 levels by two-tail test, respectively.

Meanwhile, a close relation between phenol compounds and mineral nutrients was observed as well. For example, epicatechin, and procyanidin A2 were negatively correlated with N, respectively (*p* < 0.05), and both Ca and Mg were negatively correlated with yellowish pigments like rutin, kaempferol-3-glucoside, and ferulic acid. The above indicates that mineral nutrients in the epicarp did alter the pigment profile in litchi fruit, leading to varied fruit color. Intriguingly, spraying of K salts, with the exception of K_2_SO_4_, significantly reduced epicarp Ca (*p* < 0.01). The antagonism between K versus Ca and Mg in higher plants (Garcia et al., 1999; Papadakis et al., 2004) and in litchi (Yang et al., 2015) implies that spraying of K salts might regulate the pigment composition by altering epicarp Ca and Mg, rather than by a sole and direct role of K itself. However, how the interaction between K, Ca, and Mg to affect the pigment development in litchi, needs to be further investigated.

### Differential Role of K and Mg Salts on Litchi Fruit Skin Coloration

The role of K fertilizers on anthocyanin accumulation is described in a range of plants. However, the effect is highly plant species-dependent. For example, the positive effect is observed in apple (Solhjoo et al., 2017) and olive (Zivdar et al., 2016), whereas no effect is reported in red cabbage (Piccaglia et al., 2002), purple corncob (Jing et al., 2007), and *Melastoma malabathricum* (Koay et al., 2014). In addition, a quadratic function of K fertilizer to anthocyanin is documented in batatas (Sulistiani et al., 2020) as well. The discrepancy of anthocyanin accumulation for plants might be ascribed to the varied anthocyanin composition of a specific plant *per se*.

In the present work, spray of KCl, K_2_SO_4_, and MgSO_4_ decreased the concentrations of flavanols and flavonols, but increased the levels of anthocyanins as compared to the control, indicating that anthocyanin synthesis was enhanced because these both compounds were the precursors of the latter. Unlike KCl and K_2_SO_4_, KH_2_PO_4_ spray reduced not only the values of flavanols and flavonols as well, but also the concentrations of anthocyanins. Increased synthesis of anthocyanins is a typical response of plants to P deficiency and lead to dark-brown to purple color, while P supplementation induces primary metabolism and inhibits anthocyanin synthesis by regulation of enzymes like PAL, anthocyanidin synthase, and so on, in the phenolic and flavonoid synthesis pathways (Vance et al., 2003; Misson et al., 2005). It implies that the significantly increased epicarp P by KH_2_PO_4_ spray contributes to the reduced pericarp anthocyanins in the present study.

The influence of K forms on anthocyanins is compared in a few plants. No significant difference is found on anthocyanin concentrations of purple corncobs (Jing et al., 2007) and “Red delicious” apple (Solhjoo et al., 2017) receiving K_2_SO_4_, KNO_3_, and KCl, respectively. In the current work, K_2_SO_4_ spray, superior to KCl spray enhances the production of both red pigments, implying the role of accompanying anions on anthocyanin synthesis. However, to our knowledge, the mechanism has not been investigated to date and is worthy to be revealed in the future. In addition, K_2_SO_4_ addition does not affect the concentrations of most of the phenols in litchi epicarp, but increases the levels of both red pigments by approximately 2-folds as compared to MgSO_4_ spray, highlighting the role of K^+^ on synthesis and protection of anthocyanin. The significantly decreased flavanols and flavonols and slightly increased anthocyanins by MgSO_4_ spray show that Mg treatment not only promoted anthocyanin synthesis, but also inhibited its catabolism as observed in other plants (Shaked-Sachray et al., 2002; Sinilal et al., 2011). These results refer to the importance of combination suitability of mineral cation and anion.

## Conclusion

A spray of K and Mg salts can alter the pigment profile in litchi fruit pericarp. K_2_SO_4_ spray leads to the maximum allotment of cyanidin-3-glucoside over all pigments and lower acidity in fruit epicarp, both of which jointly contributes to the highest visual color preference. This work highlights the role of spraying suitable mineral salt on improvement of fruit color and commercial value enhancement in litchi.

## Data Availability Statement

The original contributions presented in this study are included in the article/supplementary material, further inquiries can be directed to the corresponding authors.

## Author Contributions

XS: field experiment, chemical analysis, and writing of original draft. CB: phenol analysis and visual color preference evaluation on fruit. XW: field experiment. HL: field experiment and sample preparation. YZ: field experiment and sample chemical analysis. LW: photographing of litchi fruit. ZC: processing of fruit color parameters. LY: funding acquisition, methodology, field experiment, and writing – review and editing. All authors contributed to the article and approved the submitted version.

## Conflict of Interest

The authors declare that the research was conducted in the absence of any commercial or financial relationships that could be construed as a potential conflict of interest.

## Publisher’s Note

All claims expressed in this article are solely those of the authors and do not necessarily represent those of their affiliated organizations, or those of the publisher, the editors and the reviewers. Any product that may be evaluated in this article, or claim that may be made by its manufacturer, is not guaranteed or endorsed by the publisher.

## References

[B1] Agahchen A. Albu A. B. (2017). Chromatic modulation in visual art: a computational perspective. *J. Electron. Imaging* 26:011014.

[B2] Azuma A. Yakushiji H. Koshita Y. Kobayashi S. (2012). Flavonoid biosynthesis-related genes in grape skin are differentially regulated by temperature and light conditions. *Planta* 236 1067–1080. 10.1007/s00425-012-1650-x 22569920

[B3] Balcerowicz M. (2020). Tomatoes turn pale in the heat: high temperature reduces red and green pigmentation *via* phytochromes. *Plant Physiol.* 183 810–811. 10.1104/pp.20.00662 32611819 PMC7333699

[B4] Boudet A.-M. (2007). Evolution and current status of research in phenolic compounds. *Phytochemistry* 68 2722–2735. 10.1016/j.phytochem.2007.06.012 17643453

[B5] Camgoz N. Yener C. Guvenc D. (2002). Effects of hue, saturation, and brightness on preference. *Color Res. Appl.* 27 199–207. 10.1002/col.10051

[B6] Chattopadhyay T. Hazra P. Akhtar S. Maurya D. Mukherjee A. Roy S. (2021). Skin colour, carotenogenesis and chlorophyll degradation mutant alleles: genetic orchestration behind the fruit colour variation in tomato. *Plant Cell Rep.* 40 767–782. 10.1007/s00299-020-02650-9 33388894

[B7] Cubukcu E. Kahraman I. (2008). Hue, saturation, lightness, and building exterior preference: an empirical study in Turkey comparing architects’ and nonarchitects’ evaluative and cognitive judgments. *Color Res. Appl.* 33 395–405. 10.1002/col.20436

[B8] Dal Grande F. Santomaso A. Canu P. (2008). Improving local composition measurements of binary mixtures by image analysis. *Powder Technol.* 187 205–213. 10.1016/j.powtec.2008.02.013

[B9] Fang F. Zhang X. L. Luo H. H. Zhou J. J. Gong Y. H. Li W. J. (2015). An intracellular laccase is responsible for epicatechin-mediated anthocyanin degradation in litchi fruit pericarp. *Plant Physiol.* 169 2391–2408. 10.1104/pp.15.00359 26514808 PMC4677877

[B10] Fang F. Zhang Z. Q. Zhang X. L. Wu Z. X. Yin H. F. Pang X. Q. (2013). Reduction in activity/gene expression of anthocyanin degradation enzymes in lychee pericarp is responsible for the color protection of the fruit by heat and acid treatment. *J. Integr. Agric.* 12 1694–1702. 10.1016/s2095-3119(13)60410-4

[B11] Fortmann-Roe S. (2013). Effects of hue, saturation, and brightness on color preference in social networks: gender-based color preference on the social networking site Twitter. *Color Res. Appl.* 38 196–202. 10.1002/col.20734

[B12] Garcia M. Daverede C. Gallego P. Toumi M. (1999). Effect of various potassium-calcium ratios of cation nutrition of grape grown hydroponically. *J. Plant Nutr.* 22 417–425.

[B13] Green-Armytage P. (2006). The value of knowledge for color design. *Color Res. Appl.* 31 253–269. 10.1002/col.20222

[B14] Honda C. Moriya S. (2018). Anthocyanin biosynthesis in apple fruit. *Hortic. J.* 87 305–314. 10.2503/hortj.OKD-R01

[B15] Hu B. Lai B. Wang D. Li J. Q. Chen L. H. Qin Y. Q. (2019). Three LcABFs are involved in the regulation of chlorophyll degradation and anthocyanin biosynthesis during fruit ripening in *Litchi chinensis*. *Plant Cell Physiol.* 60 448–461. 10.1093/pcp/pcy219 30407601

[B16] Jezek M. Zörb C. Merkt N. Geilfus C.-M. (2018). Anthocyanin management in fruits by fertilization. *J. Agric. Food Chem.* 66 753–764. 10.1021/acs.jafc.7b03813 29297687

[B17] Jih-Gau J. Yang C. Y. (2016). Document delivery robot based on image processing and fuzzy control. *Trans. Can. Soc. Mech. Eng.* 40 677–692.

[B18] Jing P. Noriega V. Schwartz S. J. Giusti M. M. (2007). Effects of growing conditions on purple corncob (*Zea mays* L.) anthocyanins. *J. Agric. Food Chem.* 55 8625–8629. 10.1021/jf070755q 17880157

[B19] Koay S. S. Bhatt A. Shuping N. Chan L. K. (2014). Medium optimization for cell biomass yield and anthocyanin production in cell suspension culture of *Melastoma malabathricum*. *Plant Biosyst.* 148 675–682. 10.1080/11263504.2013.788569

[B20] Lee H. Wicker L. (1991). Anthocyanin pigments in the skin of lychee fruit. *J. Food Sci.* 56 466–468. 10.1111/j.1365-2621.1991.tb05305.x

[B21] Lewinsohn E. Sitrit Y. Bar E. Azulay Y. Ibdah M. Meir A. (2005). Not just colors—carotenoid degradation as a link between pigmentation and aroma in tomato and watermelon fruit. *Trends Food Sci. Technol.* 16 407–415. 10.1016/j.tifs.2005.04.004

[B22] Liu L. Xie B. Cao S. Yang E. Xu X. Guo S. (2007). A-type procyanidins from *Litchi chinensis* pericarp with antioxidant activity. *Food Chem.* 105 1446–1451. 10.1016/j.foodchem.2007.05.022

[B23] Lu R. K. (1999). *Analysis Method of Soil Agricultural Chemistry.* Beijing: China Agricultural Science and Technology Press.

[B24] Ma C. Q. Liang B. W. Chang B. Yan J. Y. Liu L. Wang Y. (2019). Transcriptome profiling of anthocyanin biosynthesis in the peel of “Granny Smith’ apples (*Malus domestica*) after bag removal. *BMC Genomics* 20:18. 10.1186/s12864-019-5730-1 31072309 PMC6507055

[B25] Misson J. Raghothama K. G. Jain A. Jouhet J. Block M. A. Bligny R. (2005). A genome-wide transcriptional analysis using *Arabidopsis thaliana* Affymetrix gene chips determined plant responses to phosphate deprivation. *Proc. Natl. Acad. Sci. U.S.A.* 102 11934–11939. 10.1073/pnas.0505266102 16085708 PMC1188001

[B26] Mizuno T. Tanaka N. Aung M. M. Yukawa T. Iwashina T. (2019). Reconstruction of flower color of *Amherstia nobilis* by *in vitro* examination. *Bull. Natl. Mus. Nat. Sci. Ser. B Bot.* 45 165–168.

[B27] Nguyen P. M. Kwee E. M. Niemeyer E. D. (2010). Potassium rate alters the antioxidant capacity and phenolic concentration of basil (*Ocimum basilicum* L.) leaves. *Food Chem.* 123 1235–1241. 10.1016/j.foodchem.2010.05.092

[B28] Paauw M. Koes R. Quattrocchio F. M. (2019). Alteration of flavonoid pigmentation patterns during domestication of food crops. *J. Exp. Bot.* 70 3719–3735. 10.1093/jxb/erz141 30949670

[B29] Papadakis I. E. Protopapadakis E. Dimassi K. N. Therios I. N. (2004). Nutritional status, yield, and fruit quality of “Encore” mandarin trees grown in two sites of an orchard with different soil properties. *J. Plant Nutr.* 27 1505–1515.

[B30] Pareek S. (2016). “Nutritional and biochemical composition of lychee (*Litchi chinensis* Sonn.) cultivars,” in *Nutritional Composition of Fruit Cultivars*, eds Simmonds M. S. J. Preedy V. R. (Cambridge, MA: Academic Press), 395–418.

[B31] Piccaglia R. Marotti M. Baldoni G. (2002). Factors influencing anthocyanin content in red cabbage (*Brassica oleracea* var *capitata* L f rubra (L) Thell). *J. Sci. Food Agric.* 82 1504–1509. 10.1002/jsfa.1226

[B32] Qu S. Li M. Wang G. Yu W. Zhu S. (2021). Transcriptomic, proteomic and LC-MS analyses reveal anthocyanin biosynthesis during litchi pericarp browning. *Sci. Hortic.* 289:110443. 10.1016/j.scienta.2021.110443

[B33] Reichel M. Carle R. Sruamsiri P. Neidhart S. (2011). Changes in flavonoids and nonphenolic pigments during on-tree maturation and postharvest pericarp browning of litchi (*Litchi chinensis* Sonn.) as shown by HPLC-MS. *J. Agric. Food Chem.* 59 3924–3939. 10.1021/jf104432r 21413696

[B34] Rodrigo M. J. Alquézar B. Alós E. Lado J. Zacarías L. (2013). Biochemical bases and molecular regulation of pigmentation in the peel of citrus fruit. *Sci. Hortic.* 163 46–62. 10.1016/j.scienta.2013.08.014

[B35] Shaked-Sachray L. Weiss D. Reuveni M. Nissim-Levi A. Oren-Shamir M. (2002). Increased anthocyanin accumulation in aster flowers at elevated temperatures due to magnesium treatment. *Physiol. Plant.* 114 559–565. 10.1034/j.1399-3054.2002.1140408.x 11975729

[B36] Sinilal B. Ovadia R. Nissim-Levi A. Perl A. Carmeli-Weissberg M. Oren-Shamir M. (2011). Increased accumulation and decreased catabolism of anthocyanins in red grape cell suspension culture following magnesium treatment. *Planta* 234 61–71. 10.1007/s00425-011-1377-0 21369922

[B37] Solhjoo S. Gharaghani A. Fallahi E. (2017). Calcium and potassium foliar sprays affect fruit skin color, quality attributes, and mineral nutrient concentrations of ‘Red Delicious’ apples. *Int. J. Fruit Sci.* 17 358–373. 10.1080/15538362.2017.1318734

[B38] Su X. X. Bai C. H. M Z. C. Zhu L. W. Yao L. X. (2022). The transfer capability of nutrient elements *via* leaf-twig-fruit in litchi. *South China Fruits* 51 75–81.

[B39] Sulistiani R. Rosmayati, Siregar L. A. M. Harahap F. (2020). The effects of temperature and potassium fertilizer on the growth, yield, and biochemical parameters of *Ipomoea batatas* var. Antin-1. *Acta Agrobot.* 73:7337. 10.5586/aa.7337

[B40] Sun J. Xiang X. Yu C. Shi J. Peng H. Yang B. (2009). Variations in contents of browning substrates and activities of some related enzymes during litchi fruit development. *Sci. Hortic.* 120 555–559. 10.1016/j.scienta.2008.12.006

[B41] Vance C. P. Uhde-Stone C. Allan D. L. (2003). Phosphorus acquisition and use: critical adaptations by plants for securing a nonrenewable resource. *New Phytol.* 157 423–447. 10.1046/j.1469-8137.2003.00695.x 33873400

[B42] Wall M. M. (2006). Ascorbic acid and mineral composition of longan (*Dimocarpus longan*), lychee (*Litchi chinensis*) and rambutan (*Nephelium lappaceum*) cultivars grown in Hawaii. *J. Food Compost. Anal.* 19 655–663.

[B43] Wilms L. Oberfeld D. (2018). Color and emotion: effects of hue, saturation, and brightness. *Psychol. Res. Psychol. Forsch.* 82 896–914. 10.1007/s00426-017-0880-8 28612080

[B44] Yang B. M. Yao L. X. Li G. L. He Z. H. Zhou C. M. (2015). Dynamic changes of nutrition in litchi foliar and effects of potassium-nitrogen fertilization ratio. *J. Soil Sci. Plant Nutr.* 15 98–110.

[B45] Yoo H. J. Kim J. H. Park K. S. Son J. E. Lee J. M. (2020). Light-controlled fruit pigmentation and flavor volatiles in tomato and bell pepper. *Antioxidants* 9:18. 10.3390/antiox9010014 31877964 PMC7023227

[B46] Zhang Z. Q. Pang X. Q. Duan X. W. Ji Z. L. Jiang Y. M. (2005). Role of peroxidase in anthocyanin degradation in litchi fruit pericarp. *Food Chem.* 90 47–52. 10.1016/j.foodchem.2004.03.023

[B47] Zhang Z. Q. Pang X. Q. Yang C. Ji Z. L. Jiang Y. M. (2004). Purification and structural analysis of anthocyanins from litchi pericarp. *Food Chem.* 84 601–604. 10.1016/j.foodchem.2003.05.002

[B48] Zhao Z. C. Hu G. B. Hu F. C. Wang H. C. Yang Z. Y. Lai B. (2012). The UDP glucose: flavonoid-3-O-glucosyltransferase (UFGT) gene regulates anthocyanin biosynthesis in litchi (*Litchi chinesis* Sonn.) during fruit coloration. *Mol. Biol. Rep.* 39 6409–6415. 10.1007/s11033-011-1303-3 22447536

[B49] Zivdar S. Arzani K. Souri M. K. Moallemi N. Seyyednejad S. M. (2016). Physiological and biochemical response of olive (*Olea europaea* L.) cultivars to foliar potassium application. *J. Agric. Sci. Technol.* 18 1897–1908.

